# Role of SIRT1 in regulation of epithelial-to-mesenchymal transition in oral squamous cell carcinoma metastasis

**DOI:** 10.1186/1476-4598-13-254

**Published:** 2014-11-26

**Authors:** I-Chieh Chen, Wei-Fan Chiang, Hsin-Hsiu Huang, Pei-Fen Chen, Ying-Ying Shen, Hung-Che Chiang

**Affiliations:** Division of Environmental Health and Occupational Medicine, National Health Research Institutes, Miaoli, Taiwan; Department of Oral & Maxillofacial Surgery, Chi-Mei Medical Center, Liouying, Tainan, Taiwan; School of Dentistry, National Yang-Ming University, Taipei, Taiwan; Pathology Core Laboratory, National Health Research Institutes, Miaoli, Taiwan; National Environmental Health Research Center, National Health Research Institutes, Miaoli, Taiwan; Department of Occupational Medicine, Taipei Medical University-Shuang Ho Hospital, Taipei, Taiwan

**Keywords:** Sirtuin 1, Epithelial-to-mesenchymal transition, Migration, Metastasis, Matrix metalloproteinase-7, Oral squamous cell carcinoma

## Abstract

**Background:**

The epithelial-to-mesenchymal transition (EMT) process results in a loss of cell-cell adhesion, increased cell mobility, and is crucial for enabling the metastasis of cancer cells. Recently, the enzyme SIRT1 has been implicated in a variety of physiological processes; however, its role in regulating oral cancer metastasis and EMT is not fully elucidated. Here, we propose a mechanism by which the enzyme sirtuin1 (SIRT1) regulates the EMT process in oral cancer by deacetylating Smad4 and repressing the effect of TGF-β signaling on matrix metalloproteinase-7 (MMP7).

**Methods:**

The roles of SIRT1 in tumor cell migration/invasion and metastasis to the lungs were investigated using the Boyden chamber assay and orthotopic injections, respectively. RNA interference was used to knockdown either SIRT1 or Smad4 expression in oral squamous cell carcinoma (OSCC) cell lines. Immunoblotting, zymographic assays, and co-immunoprecipitation were used to examine the effects of SIRT1 overexpression on MMP7 expression and activity, as well as on SIRT1/ Smad4 interaction.

**Results:**

We found that compared with normal human oral keratinocytes (HOKs), SIRT1 was underexpressed in OSCC cells, and also in oral cancer tissues obtained from 14 of 21 OSCC patients compared with expression in their matched normal tissues. Overexpression of SIRT1 inhibited migration of OSCC cells *in vitro*, as well as their metastasis to the lung *in vivo*. Furthermore, up-regulation of SIRT1 in metastatic OSCCs significantly inhibited the migration and invasion abilities of OSCC cells, while concomitantly increasing the expression of E-cadherin, and decreasing the expressions of mesenchymal markers. We also identified Smad4, a TGF-β-activated transcription factor, as a direct target protein for SIRT1. Overexpression of SIRT1 in OSCC cells led to decreased levels of acetylated Smad4, and inhibition of TGF-β-induced signaling. By associating and deacetylating Smad4, SIRT1 enzyme can influence MMP7 expression, MMP enzyme activity, and consequently, cell migration, invasion, and tumor metastasis in OSCCs.

**Conclusions:**

These findings provide a valuable insight into the potential role of the SIRT1 enzyme in regulating cell migration and invasion in oral squamous cell carcinoma. Our findings suggest the SIRT1/Smad4/MMP7 pathway as a target for oral cancer driven by EMT.

**Electronic supplementary material:**

The online version of this article (doi:10.1186/1476-4598-13-254) contains supplementary material, which is available to authorized users.

## Background

Oral cancer is the sixth most common human cancer worldwide, and >90% of oral malignancies are squamous cell carcinomas [1]. Oral squamous cell carcinoma (OSCC) accounts for >95% of all head and neck cancers, and can develop from oral precancerous lesions such as leukoplakia and erythroplakia [1–3]. The incidence of oral cancer in Taiwan has increased 30% during the last 5 years, and the overall mortality rate has increased 25%. Males aged 30–49 years have the highest rate of mortality due to oral cancer [4, 5]. More than 50,000 new cases of oral cancer are diagnosed annually, and the overall 5-year survival rate for OSCC patients during the last 2 decades has consistently remained between 34% and 62.7% [5–7]. It was recently reported that the cervical lymph node is a critical prognostic indicator of the clinical course of OSCC, and that patients with cervical lymph node metastasis usually have lower survival rates [8–10]. Similar to other cancers, oral cancer metastasis occurs after a localized tumor progresses to an advanced stage [11]. Therefore, an understanding of the molecular mechanism which regulates OSCC metastasis can provide information important for developing new drugs and guidelines for treating metastasized oral cancers. Cancer metastasis is accelerated by an epithelial to mesenchymal transition (EMT) process resulting in increased cell migration and invasion, cell-substrate adhesion, intravasation and extravasation, as well as increased cell survival. EMT plays an important role in cancer invasion and metastasis, during which epithelial cells lose their cell-adhesive properties, repress E-cadherin expression, and increase their levels of mobility, matrix metalloproteinases (MMPs), and expression of mesenchymal markers [12–14]. E-cadherin is a cell-cell adhesion molecule expressed predominantly by epithelial cells. Reduction or loss of E-cadherin is considered a hallmark event of EMT, which initiates a series of signaling events and a major reorganization of the cell cytoskeleton [15, 16]. Concomitant with the loss of E-cadherin and actin reorganization, cells undergoing EMT acquire a mesenchymal phenotype that becomes apparent by the expression of mesenchymal cytoskeletal proteins such as vimentin, and increased deposition of extracellular matrix proteins by MMPs. These extracellular matrix components stimulate integrin signaling and facilitate cell migration [17, 18]. Furthermore, decreased expression of E-cadherin during EMT is accompanied by increased expression of N-cadherin, which renders the cell more motile and invasive [19–21]. These different events result in a loss of apical-basal polarity, after which, the cells acquire a front-back polarity that allows them to migrate in a directional fashion. The increased MMP expression and activity allows the cells to degrade extracellular matrix proteins, permitting their delamination and escape from their epithelial components [22]. In cancer, epithelial tumor cells become more invasive after undergoing EMT, and enter the circulatory system through intravasation. This results in their dissemination to loci distal from the primary tumor. Hence, elucidating the molecular mechanism which regulates expression of E-cadherin, N-cadherin, and MMPs, has become pivotal for understanding cancer invasion and metastasis.

Sirtuins are nicotinamide adenine dinucleotide (NAD^+^)-dependent histone deacetylases [23]. Human homologues of the *Sir2* gene are found in yeast, and are considered a critical link to longevity, as they prolong the cellular replication cycles of *Saccbaromyces Cerevisiae* and *Caenorbabditis elegans*[24, 25]. Several types of sirtuin enzymes have been identified (SIRT1-7), and their enzymatic activities are regulated by the ratio of NAD^+^ to NADH; high NAD^+^ levels activate sirtuin enzymes, and conversely, high NADH levels inhibit their activity [26]. Due to their abilities to deacetylate both histone and non-histone substrates, sirtuin enzymes have roles in regulating multiple cellular and physiological processes, including diabetes, inflammation, neurodegenerative diseases, stress responses, cell survival, metabolism, aging, and longevity [27–30]. Sirtuin enzymes are widely expressed in normal tissues. SIRT1 localizes primarily in the nucleus, along with SIRT6 and SIRT7; whereas SIRT2 is in the cytoplasm, and SIRT3, SIRT4, and SIRT5 are localized in the mitochondria [31].

SIRT1 is a class III histone deacetylase capable of deacetylating lysine residues on nuclear proteins, which is thought to affect their stability, transcriptional activity, and translocation. Recently, SIRT1-mediated deacetylation of nuclear proteins such as p53, FOXO, and Ku70 [32–34], has been reported to promote cell survival. Roles for SIRT1 in skin, colon, breast, and lung cancers have been demonstrated through its affects on one or more of the aforementioned nuclear proteins [35–39]. Additionally, SIRT1 can regulate vascular endothelial homeostasis by controlling angiogenesis and vascular function [40], and also regulates the transcription of numerous genes by interacting with transcription factors. For example, upon recruitment to chromatin by transcription factors, SIRT1 deacetylates histones to suppress gene transcription [28, 41, 42]. Despite evidence for SIRT1 involvement in a variety of cell regulatory and physiological processes, the role of SIRT1 in regulating oral cancer metastasis and EMT remains enigmatic. In this study, we investigated the involvement of SIRT1 in EMT as it occurs in oral cancer metastasis. We found that SIRT1 expression was substantially downregulated in OSCC cell lines, and was also widely attenuated in OSCC tumors as compared with expression in paired normal tissues. SIRT1 overexpression repressed the EMT process in oral cancers and blocked migration of OSCC cells *in vitro*. In contrast, knockdown of SIRT1 in oral cancer cells enhanced EMT and cancer metastasis *in vitro*. We also show that SIRT1 regulates expression of the epithelial marker E-cadherin, as well as the mesenchymal markers vimentin and N-cadherin. Moreover, we found that SIRT1 targets Smad4 to reduce EMT and MMP7 expression. Finally, we show that SIRT1 overexpression reduced the invasiveness and metastasis of oral cancer cells in immunodeficient mice. In summary, our data show that SIRT1 inhibited the EMT process in oral cancer by deacetylating Smad4 and repressing expression of MMP7. These results suggest a role for SIRT1 as a metastasis suppressor in oral cancer.

## Results

### Variable levels of SIRT1 expression and its activity

**Figure 1:**
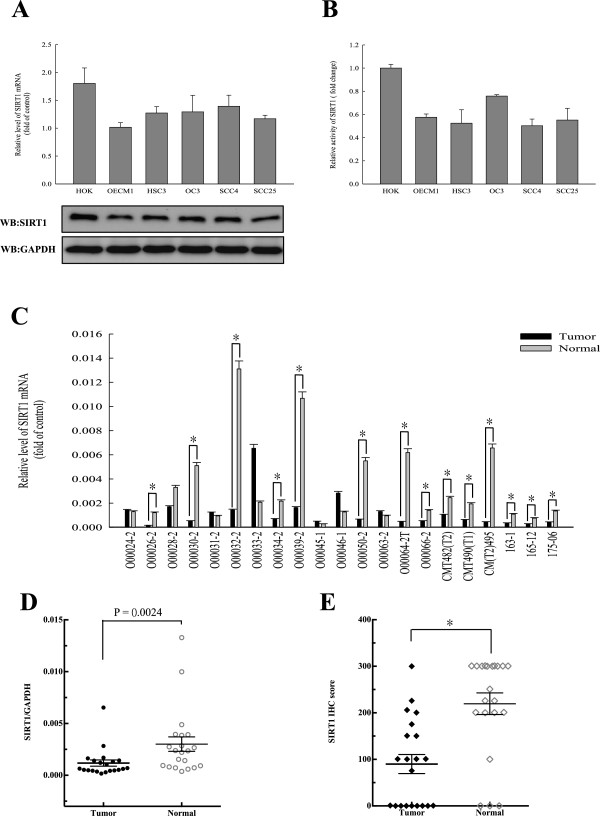
**Variable levels of SIRT1 expression and its activity were noted among normal cells (HOK) and OSCCs. (A)** Quantitative RT-PCR (qRT-PCR) and western blotting revealed the expression levels of SIRT1 in HOK and OSCC cell lines. **(B)** Specific activities of SIRT1 in HOK and OSCC cell lines were determined by enzyme assays. Equal amounts of cellular SIRT1 protein (250 ng) were immunopurified with antibodies against SIRT1, and SIRT1 enzyme activity assays were performed with a SIRT1 Fluorometric Kit, using standard protocols provided by the supplier. **(C and D)** qRT-PCR revealed significant underexpression (P = 0.0024 ) of SIRT1 in 14 of 21 OSCC samples compared with their matched normal tissues. **(E)** The expression levels of SIRT1 in the normal and tumor tissues of 21 OSCC patients as determined by IHC. The IHC semi-quantitative score was derived by two independent pathologists who multiplied the staining intensity by the percent of tumor cells stained. IHC scores for each core of a specimen were averaged (n = 21) and statistically analyzed (*, p <0.05). Each data point represents the mean value ± SD obtained from at least three independent experiments.

To evaluate the role of SIRT1 in regulating oral cancer metastasis and EMT, we first investigated whether SIRT1 expression in normal primary human oral keratinocytes (HOKs) differed from that in OSCC cells. We examined the SIRT1 mRNA and protein levels in 5 OSCC cell lines (HSC3, OECM1, OC3, SCC4, and SCC25) and compared them with their levels in HOK cells (Figure 1A). We found that both the transcription and translation products of SIRT1 were more highly expressed in HOKs compared to their expressions in various OSCC cell lines. Next, we isolated the nuclear fractions of HOK cells and OSCC cells, immunoprecipitated the endogenous SIRT1, and tested for its deacetylase activity. Surprisingly, we found that all OSCC cell lines had drastically lower levels (~50%) of SIRT1 activity compared with those in HOK cells (Figure 1B). Additionally, we examined 21 pairs of oral normal and cancer tissues obtained from OSCC patients and tested them for SIRT1 mRNA expression. We found that SIRT1 mRNA levels were drastically underexpressed (P = 0.0024) in 14 of the 21 OSCC samples compared with expression in their matched normal tissues (Figure 1C and D). We next used immunohistochemistry (IHC) techniques to analyze the levels of SIRT1 expression in clinical samples. We found that 15 pairs of matched normal and tumor tissue samples obtained from 21 OSCC patients showed significantly higher SIRT1 expression in the normal tissue as compared to the tumor tissue (Figure 1E). These results suggested that SIRT1 might exclusively be responsible for the development of oral cancer, and that decreasing SIRT1 expression and enzyme activity may increase an individual’s susceptibility to tumorigenesis and metastasis of oral cancer.

### SIRT1 represses migration and invasion of OSCC cells through its deacetylase activity

**Figure 2:**
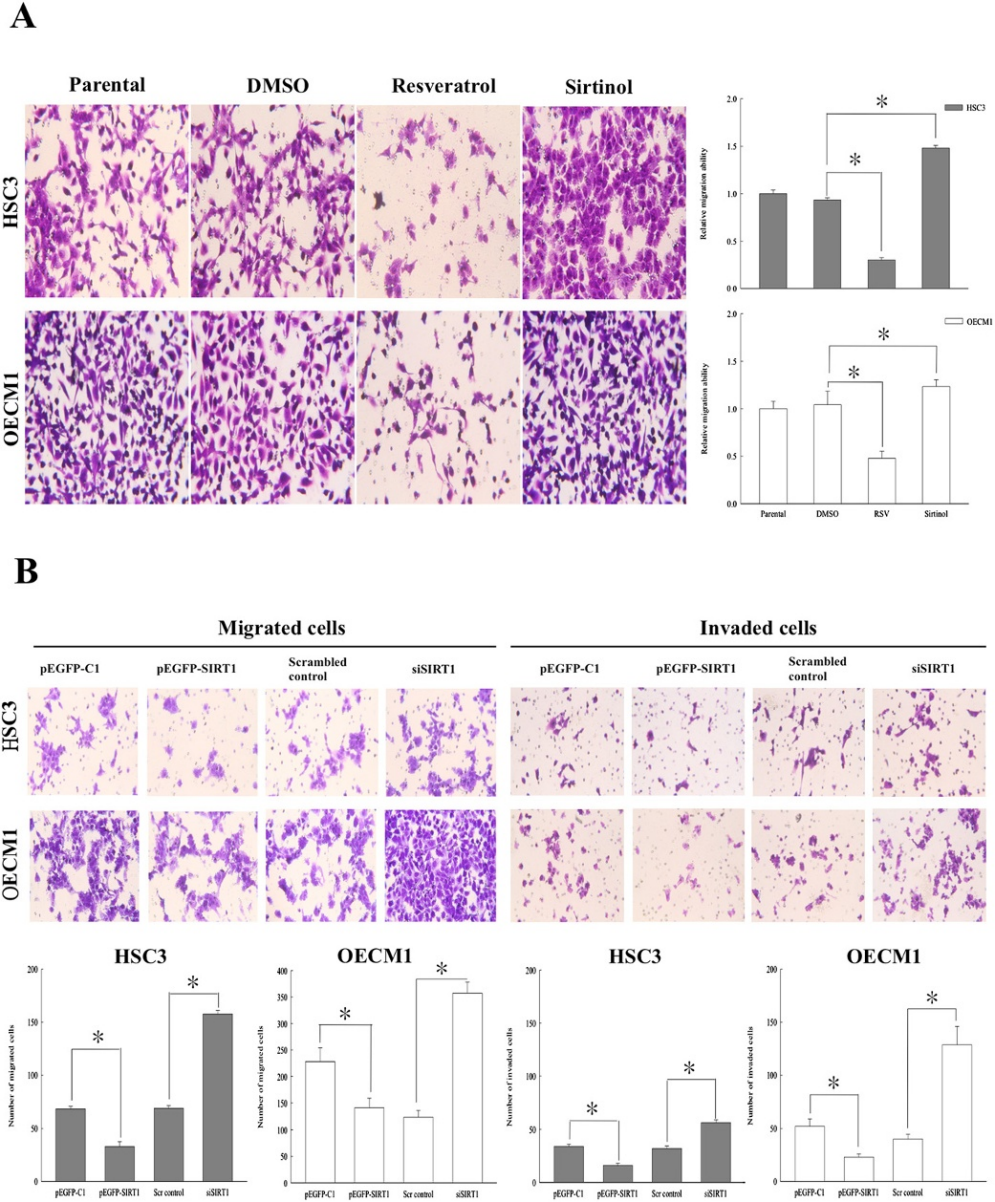
**SIRT1 activation prevents oral cancer metastasis. (A)** OSCC cells (10^5^) were treated with 50 uM resveratrol (RSV; an SIRT1 agonist) and 10 uM sirtinol (an SIRT 1 antagonist) for 24 h, respectively. **(B)** Transient transfection of pEGFP-SIRT1 significantly inhibited the migration and invasion of OECM1 and HSC3 cells, which were rescued by siSIRT1. Transient transfected cells (overexpression-SIRT1 or knockdown SIRT1) were seeded in a 24-well chemotaxis chamber (1 × 10^4^ cells/well) and incubated for 24 h with complete culture medium added in the lower chamber. Cell migration and invasion by Boyden chamber assays. Each data point represents the mean ± SD from at least three independent experiments. The asterisk indicates as statistically significant difference (*, p <0.05) compared to the pEGFP-C1 vector control or scrambled siRNA control.

SIRT1 is a histone/protein deacetylase, and numerous studies have reported SIRT1 involvement in the regulation of various processes through its deacetylase activity [41]. Therefore, we conducted Boyden Chamber assays to determine whether the deacetylase activity of SIRT1 would suppress the migration and invasion of oral cancer cells. As expected, activation of SIRT1 in OSCC cell lines by resveratrol (RSV; a SIRT1 agonist) suppressed the migration of OECM1 and HSC3 cells. In contrast, an SIRT1 antagonist (sirtinol) was completely ineffective in suppressing cell migration, and greatly increased oral cancer cell metastasis *in vitro* (Figure 2A). Next, we ectopically expressed SIRT1 in OSCC cell lines OECM1 and HSC3, thus taking advantage of their low SIRT1 expression. As shown in Figure 2B, overexpression of SIRT1 induced by transient transfection significantly blocked the migration and invasion of OSCC cells, as compared with the migration and invasion behaviors shown by pEGFP-C1 vector only transfected control cells. Furthermore, we also knocked down SIRT1 expression in both OSCC cell lines with or without siRNA oligonucleotides, and found that knockdown cells displayed significantly increased migration and invasion abilities (p <0.05), compared with those shown by Scrambled control cells. These results indicated that the migration and invasion of OSCC cells were significantly suppressed by exogenous overexpression of SIRT1, while repression of SIRT1 by small interfering RNA molecules increased the metastatic potential of OSCC cells. Thus, SIRT1 activation appears to be tightly correlated with cell migration and invasion ability, and SIRT1 might be an important regulator of migration and invasion in oral cancer cells.

### SIRT1 regulates expression of epithelial and mesenchymal protein markers

**Figure 3:**
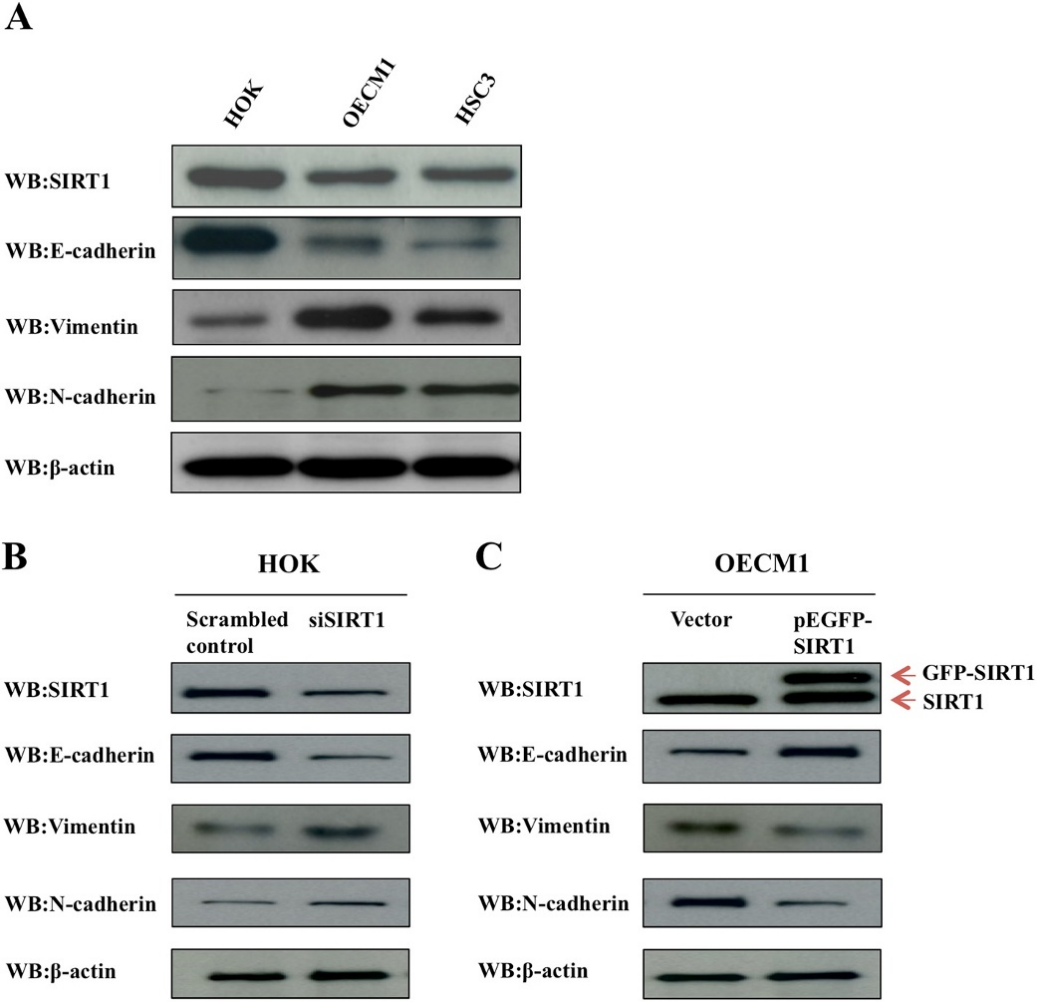
**Expression of epithelial and mesenchymal protein markers are regulated by SIRT1 in HOK and OSCC cells. (A)** Western blotting revealed the expression levels of epithelial and mesenchymal protein markers in HOK and OSCC cell lines. Equal amounts of cell protein (20 ug) were immunoblotted with antibodies against SIRT1, E-cadherin, vimentin, N-cadherin, and β-actin. **(B)** Loss of SIRT1 increased expression of endogenous mesenchymal protein markers. Western blotting revealed expression of SIRT1 and EMT markers in HOK cells with or without siSIRT1. **(C)** Ectopic expression of SIRT1 increased expression of E-cadherin and reduced expression of vimentin and N-cadherin. Equal amounts of protein (20 ug) from OECM1 cells were transient transfected with pEGFP-SIRT1 or vector alone (pEGFP-C1) and analyzed by Western blot.

Previous studies have described E-cadherin as a well-established hallmark of EMT [14]. Therefore, we sought to determine whether E-cadherin expression is altered in OSCC cell lines. Surprisingly, we found that SIRT1 and E-cadherin were overexpressed in HOK cell lines compared to their expression in both OSCC cell lines. In contrast, SIRT1, as well as mesenchymal marker proteins N-cadherin and vimentin, were inversely expressed at the basal condition in normal HOK cells, and also in the OSCC cell lines OECM1 and HSC3 (Figure 3A). We next investigated the possible regulation of E-cadherin, N-cadherin, and vimentin expression by SIRT1, by using siRNA oligonucleotides to knock down SIRT1 expression in HOK cell lines, and found that SIRT1 silencing clearly down-regulated E-cadherin expression. Additionally, the deletion of SIRT1 led to significantly increased N-cadherin and vimentin expression in knockdown HOK cells. A similar reciprocal relationship was observed in the case of SIRT1 overexpression in OECM1 cells, which showed increased E-cadherin expression (Figure 3B and C). Moreover, we also determined the expression of certain mesenchymal markers important for EMT. Transfection of OSCC cells with an SIRT1 expression vector resulted in SIRT overexpression which subsequently reduced the expression of the mesenchymal proteins N-cadherin and vimentin. Together, these data indicated that SIRT1 may play a role in regulating epithelial and mesenchymal protein expression.

### SIRT1 represses expression of MMP7 in OSCC cells

**Figure 4:**
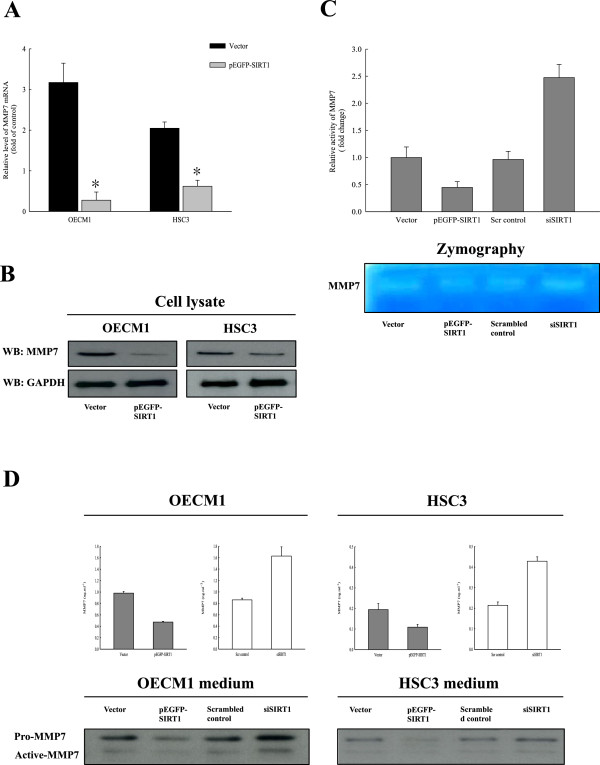
**SIRT1 down-regulates expression and activity of MMP7. (A)** qRT-PCR revealed the expression levels of MMP7 in OSCC cell lines after transient transfection with pEGFP-SIRT1 or vector alone (pEGFP-C1). Each data point represents the mean ± SD from at least three independent experiments. The asterisk indicates statistically significant difference (*, p <0.05) comparing to the control. **(B)** The MMP7 protein level was assessed by immunoblotting with anti-MMP7 antibody and GAPDH. MMP7 proteins in the cell homogenates were evaluated. **(C)** MMP7 activity was assayed by casein zymography. MMP7 activities in the cell media were compared between SIRT1-overexpressing or silencing OECM1 cells. **(D)** MMP7 concentrations and protein levels in OSCC cell media were assessed by ELISA and immunoblotting with anti-MMP7 antibody and GAPDH. Cell culture media were collected and concentrated from SIRT1-overexpressing or silencing OSCC cell lines.

Similar to the metastatic mechanism of other cancers, oral cancer metastasis requires an extensive remodeling and degradation of the extracellular matrix, partially via increased expression of matrix metalloproteinases (MMPs) [43]. MMP7 expression has been significantly correlated with oral cancer metastasis and EMT [44, 45], which suggests that the SIRT1 overexpression might affect MMP7 expression in OSCCs. We thus examined the effect of transiently expressed SIRT1 on OSCC cell lines by using a GFP-tagged SIRT1 expressing vector. We found that *MMP7* transcription and translation were significantly decreased in SIRT1-overexpressing cells compared with their levels in control cells (Figure 4A and B). We also compared the enzymatic activity of MMP7 in SIRT1 overexpressing and silencing OSCC cells. When MMP7 activity was assayed by casein zymography, the activity in the media from SIRT1-overexpressing OECM1 cells was significantly lower than that in media from mock-transfected cells. In contrast, SIRT1 silencing produced a significant increase in MMP7 activity (Figure 4C). This activity change is probably due to the difference in the protein levels, as determined by ELISA and immunoblotting with anti-MMP7 antibody. The levels of MMP7 secreted into the media of OSCC cell lines were also estimated by ELISA at 48 h after transfection with a SIRT1 expression vector or siSIRT1. We found that MMP7 secretion by SIRT1-overexpressing OSCC cells was significantly suppressed (p <0.05) as compared with secretion by mock-transfected cells. In contrast, SIRT1 silencing in oral cancer cells resulted in a significant induction of MMP7 secretion. A similar result was seen in western blot experiments, where MMP7 secretion was significantly suppressed by exogenously produced overexpression of SIRT1 in both OSCC cell lines, whereas repression of SIRT1 by SIRT1 silencing increased MMP7 secretion (Figure 4D). We further studied the mechanism by which SIRT1 regulates MMP7 expression by determining whether SIRT1 could associate and deacetylate MMP7. Immunoprecipitations performed with an anti-SIRT1 or anti-MMP7 antibody in OSCC cells failed to identify any endogenous molecular binding between SIRT1 and MMP7 (data not shown). This result indicated that SIRT1 could influence MMP7 expression, secretion, and activity; and subsequently, cell migration, invasion, and metastasis through its target proteins.

### SIRT1 deacetylates Smad4 in OSCC cells

**Figure 5:**
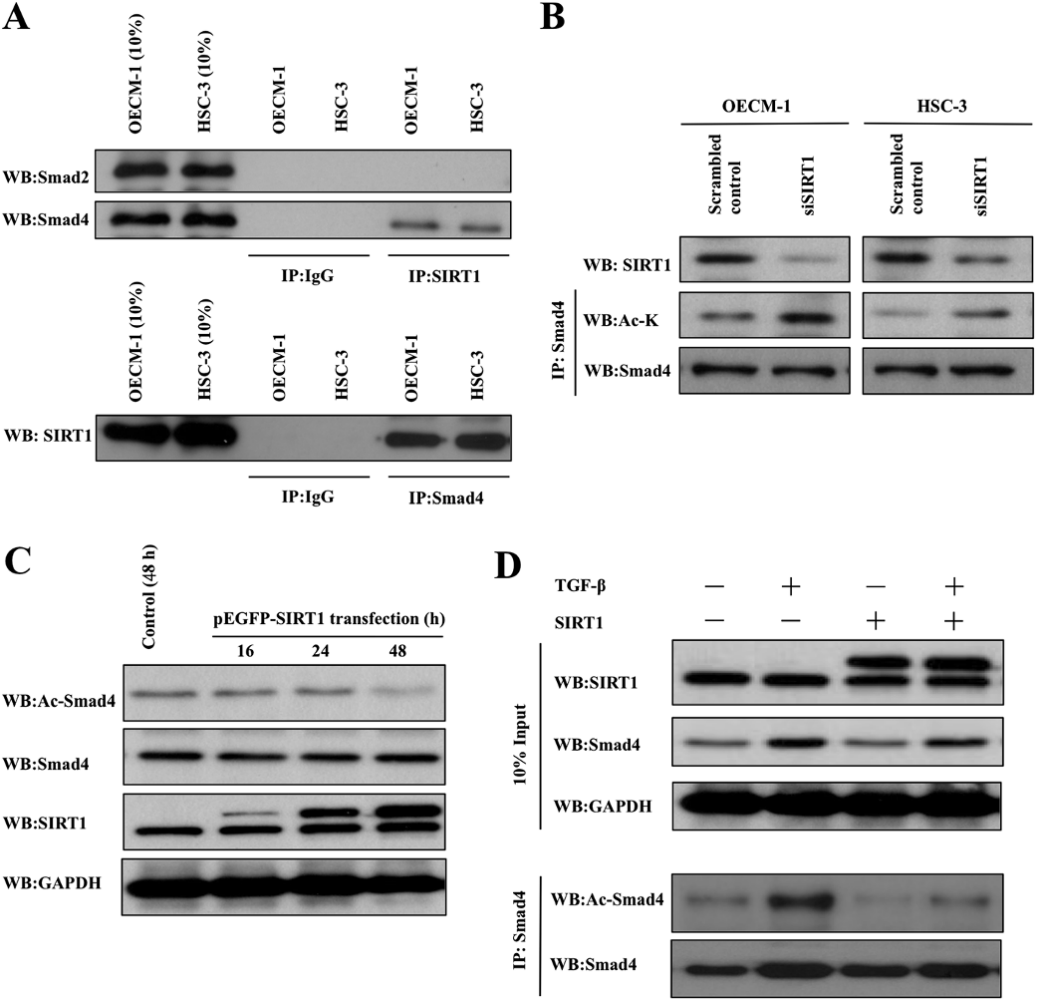
**SIRT1 interacts with Smad4. (A)** Smad4 binds to SIRT1 in OSCC cells. Nuclear extracts from OECM1 and HSC3 cells were immunoprecipitated using a SIRT1 or Smad4 antibody, and analyzed by western blot using antibodies against Smad2, Smad4, and SIRT1. **(B)** Knock down of SIRT1 increased endogenous Smad4 acetylation. Acetylated endogenous Smad4 proteins in OSCC cells with or without siSIRT1 were then immunopurified with Smad4 antibody. Western blots were probed with SIRT1, acetylated-Lysine (Ac-K), and Smad4. **(C)** Ectopic expression of SIRT1 reduced levels of acetylated Smad4. Equal amounts of protein (20 ug) from OECM1 cells were transient transfected with pEGFP-SIRT1 or vector alone (pEGFP-C1) for 0–48 h and analyzed by Western blot. **(D)** Western blotting revealed the expression and acetylation levels of endogenous Smad4 in OECM1 cell lines transient transfected with pEGFP-SIRT1 or vector alone (pEGFP-C1) for 24 h, and treated with TGF-β 5 ng/mL for 48 h. Western blots were probed with SIRT1, acetylated-Lysine (Ac-K), and Smad4.

MMP7 has been shown to be important for accelerating cancer invasion and metastasis in multiple tissues [46], but does not seem to be necessary for invasion or fibrosis of colon cancer, in which Smad4-dependent transforming-growth-factor (TGF)-β family signaling is blocked [47]. Thus MMP7 is not needed for tissue invasion in Smad4-deficient adenocarcinomas. Additionally, a previous study revealed that SIRT1 directly interacts with and deacetylates the negative regulator of TGF-β signaling, Smad7, to destabilize the protein in a mesangial kidney cell line [48]. We therefore postulated that SIRT1 might affect MMP7 through its interactions with Smad4, a TGF-β-activated transcription factor. To test this hypothesis, we first used an immunoprecipitation assay to examine the ability of SIRT1 to bind to Smad4. Our results showed that while SIRT1 directly interacted with Smad4 *in vivo*, it did not interact with Smad2 protein (Figure 5A). We also performed a co-immunoprecipitation experiment to examine the ability of Smad4 to bind SIRT1. Western blotting detected SIRT1 in the Smad4 immunoprecipitate from nuclear extracts of OSCCs. We next examined whether SIRT1 could directly deacetylate Smad4. We immunopurified endogenous Smad4 from SIRT1 knockdown OECM1 and HSC3 cells, and probed western blots with antibodies to Smad4 proteins or acetylated-lysine (Figure 5B). This experiment showed that SIRT1 silencing significantly increased the level of acetylated Smad4 in SIRT1 knockdown OSCC cells. Furthermore, we also confirmed the acetylation levels of Smad4 in OECM1 and HSC3 cells at 0, 16, 24, and 48 h after transfection with the SIRT1 expression vector. Overexpression of SIRT1 clearly reduced the acetylation levels of Smad4, while knockdown of SIRT1 increased the acetylation levels (Figure 5C and Additional file 1: Figure S1A). These results suggest that while SIRT1 associates with and deacetylates Smad4, the SIRT1 deacetylase activity is not required for Smad4 protein expression.

Because Smad4 is a transcription factor that responds to TGF-β signaling, we next investigated the expression levels of Smad4 in SIRT1-overexpressing OECM1 and HSC3 cells following TGF-β stimulation (Figure 5D and Additional file 1: Figure S1A). We observed that levels of endogenous Smad4 protein in SIRT1-overexpressing or mock-transfected cells were increased ~2-fold after 48 h of TGF-β stimulation. Surprisingly, the acetylation level of Smad4 was highly increased by TGF-β induction, while overexpression of SIRT1 significantly reduced the acetylation level of TGF-β induced Smad4 in OSCCs. Taken together, suggest that SIRT1 functionally interacts with Smad4 *in vivo*, resulting in the deacetylation of Smad4 and inhibition of TGF β-induced signaling.

### SIRT1 regulates MMP7 expression through deacetylating Smad4

**Figure 6:**
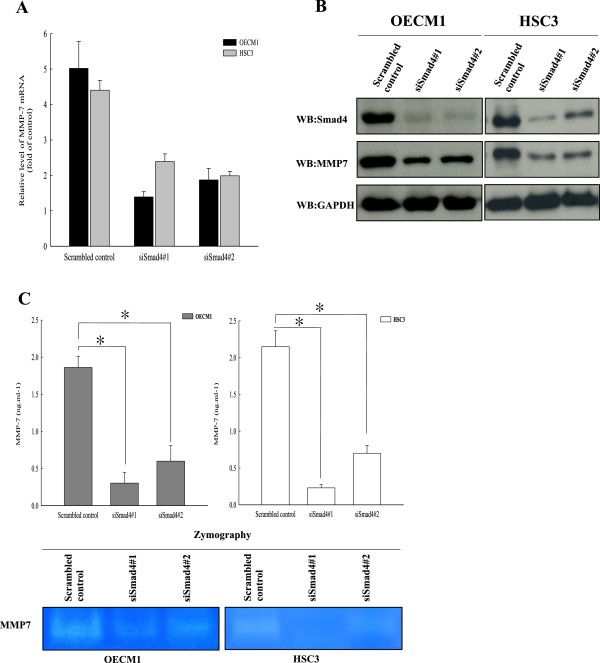
**Smad4 regulates MMP7 expression in OSCCs. (A)** Quantitative RT-PCR expression of the MMP-7 gene in OECM1 and HSC3 cell lines with or without siSmad4. **(B)** Western blotting revealed the expression levels of MMP7 in OSCC cells with or without siSmad4. **(C)** MMP7 concentrations and activity in OECM1 and HSC3 media were assessed by ELISA and casein zymography, respectively. Cell culture media were collected and concentrated from Smad4 silencing OSCC cell lines. Each data point represents the mean ± SD of at least three independent experiments. The asterisk indicates as statistically the significant difference (*, p <0.05) compared to control.

**Figure 7:**
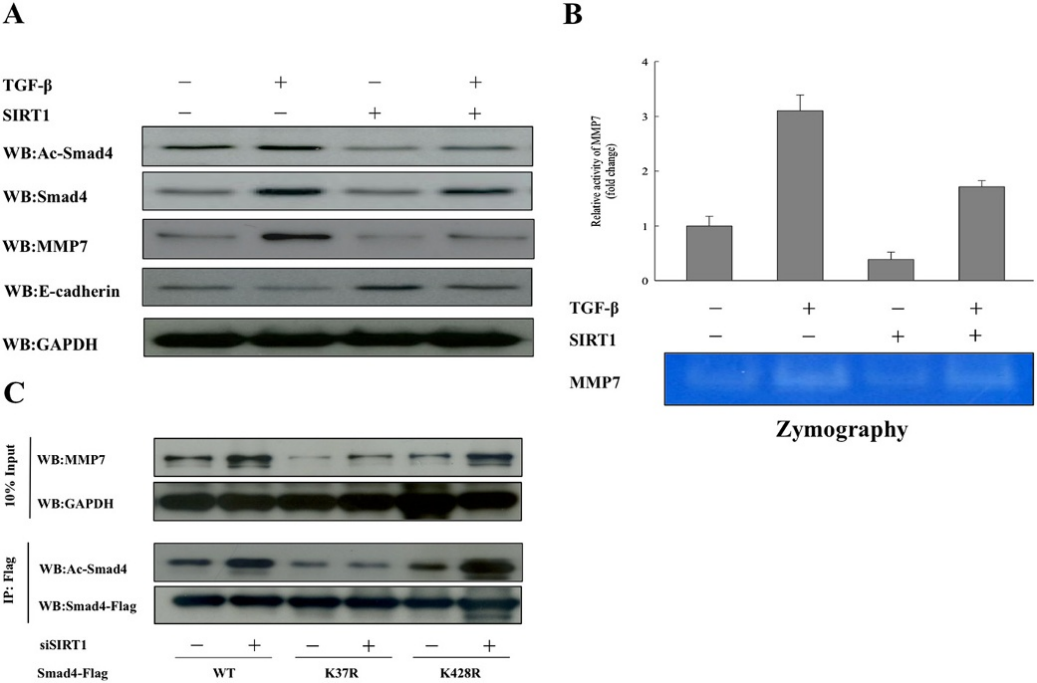
**SIRT1 represses the expression of MMP7 by deacetylating Smad4 in OSCCs. (A)** Western blotting reveled the expression levels of Smad4, MMP7, and E-cadherin in OECM1 cell lines transient transfected with pEGFP-SIRT1 or vector alone (pEGFP-C1) for 24 h, and treated with TGF-β (5 ng/mL) for 48 h. **(B)** MMP7 activities of SIRT1-overexpressing or mock-transfected OECM1 cells were assayed by casein zymography after treatment with or without (TGF-β 5 ng/mL) for 48 h. **(C)** SIRT1 repressed expression of MMP7 by deacetylating K37 of Smad4. Western blotting revealed the expression and acetylation levels of endogenous Smad4 from flag-tagged Smad4 WT, Smad4-K37R, and Smad4-K428R OECM1 mutants with or without siSIRT1, and then purified by Flag beads. Western blots were probed with flag, acetylated-Lysine (Ac-K), and MMP7.

Previous studies have suggested that Smad4 may regulate MMP7 expression in cancer, and we therefore examined the effect of transiently silencing Smad4 in oral squamous carcinoma cells by transfected siRNA. Our results showed that MMP7 mRNA expression reduced, and a similar result was seen in a Western blot experiment. SIRT1 silencing significantly downregulated MMP7 protein expression in both OSCC cell lines (Figure 6A and B). We then collected and concentrated cell culture media from Smad4-silencing cells. A subsequent ELISA analysis of the media showed that MMP7 secretion was significantly decreased in siSmad4 OSCC cells compared with secretion in scrambled control OSCC cells. Assays of MMP7 concentrations and activity by casein zymography and ELISA revealed that MMP7 activity in the media from the siSmad4 OECM1 and HSC3 cells was significantly lower than that in the media of control cells, and a similar result was shown by studies of MMP7 concentration (Figure 6C). These experiments showed that Smad4 regulates and is required for MMP7 expression, secretion, and activity in oral cancer. To address whether the SIRT1 regulation of MMP7 expression was modulated via the TGF-β transcription factor Smad4, we monitored MMP7 expression in SIRT1-overexpressing OECM1 and HSC3 cells following their stimulation with TGF-β. As shown in Figure 7A and Additional file 2: Figure S2A, TGF-β stimulation increased Smad4 expression and hyperacetylation of Smad4 in both OSCC cell lines. Additionally, TGF-β also induced expression of MMP7, which became hyperexpressed when Smad4 was hyperacetylated following TGF-β stimulation. Next, we ectopically expressed SIRT1 in OECM1 and HSC3 cell lines, and found that overexpression of SIRT1 in OSCC cells led to both decreased levels of Smad4 acetylation, and repressed affects of TGF-β signaling on MMP7. TGF-β induces MMP7 expression which results in extracellular cleavage of E-cadherin from the cell surface, and disruption of E-cadherin [49, 50]. Therefore, we also tested the effect of E-cadherin expression in SIRT1-overexpressing cells after they had been pre-treated with TGF-β. Interestingly, while TGF-β reduced E-cadherin levels in both mock-transfected cells and SIRT1-overexpressing OSCC cells, the reductions were much greater in SIRT1-overexpressing cells. Similarly, MMP7 activity in mock-transfected cells was markedly increased by TGF-β stimulation (Figure 7B and Additional file 2: Figure S2B). In contrast, overexpression of SIRT1 in oral cancer cells caused a significant reduction of MMP7 activity, while TGF-β stimulation was slightly reversed the increase in MMP7 activity (p <0.05). This change was closely related to the deacetylation levels of Smad4, and might be responsible for the reduced efficiency of TGF-β signaling in regulating MMP7 expression. Recently, several acetylation sites in Smad4 isolated from the nucleus have been identified by high-resolution mass spectrometry [51]. However, the acetylation site in Smad4 which directly interacts with SIRT1 remains unknown. We generated a flag-tagged Smad4 WT, Smad4-K37R, and Smad4-K428R mutant OECM1 cells, and analyzed their acetylation levels. After immunopurifying ectopically expressed Flag-tagged Smad4 proteins from OECM1 mutants after knock down of SIRT1, we found that the acetylation mimetic mutant Smad4^K37R^ had a significantly decreased level of acetylation compared to the wild-type Smad4. Whereas K428R substitution greatly increased acetylation to levels similar to those observed in wild-type Smad4 (Figure 7C). Together, these observations indicated that TGF-β stimulation increased Smad4 and MMP7 expression, and SIRT1 deacetylated Smad4 *in vivo*; additionally, K37 was the primary target of SIRT1, resulting in decreased MMP7 expression and activity. Thus, SIRT1 participates in regulation of MMP7 activity and expression by deacetylating K37 of Smad4, and repressing the effect of TGF-β signaling in oral cancer.

### Overexpression of SIRT1 inhibits lung metastasis of OSCC cells

**Figure 8:**
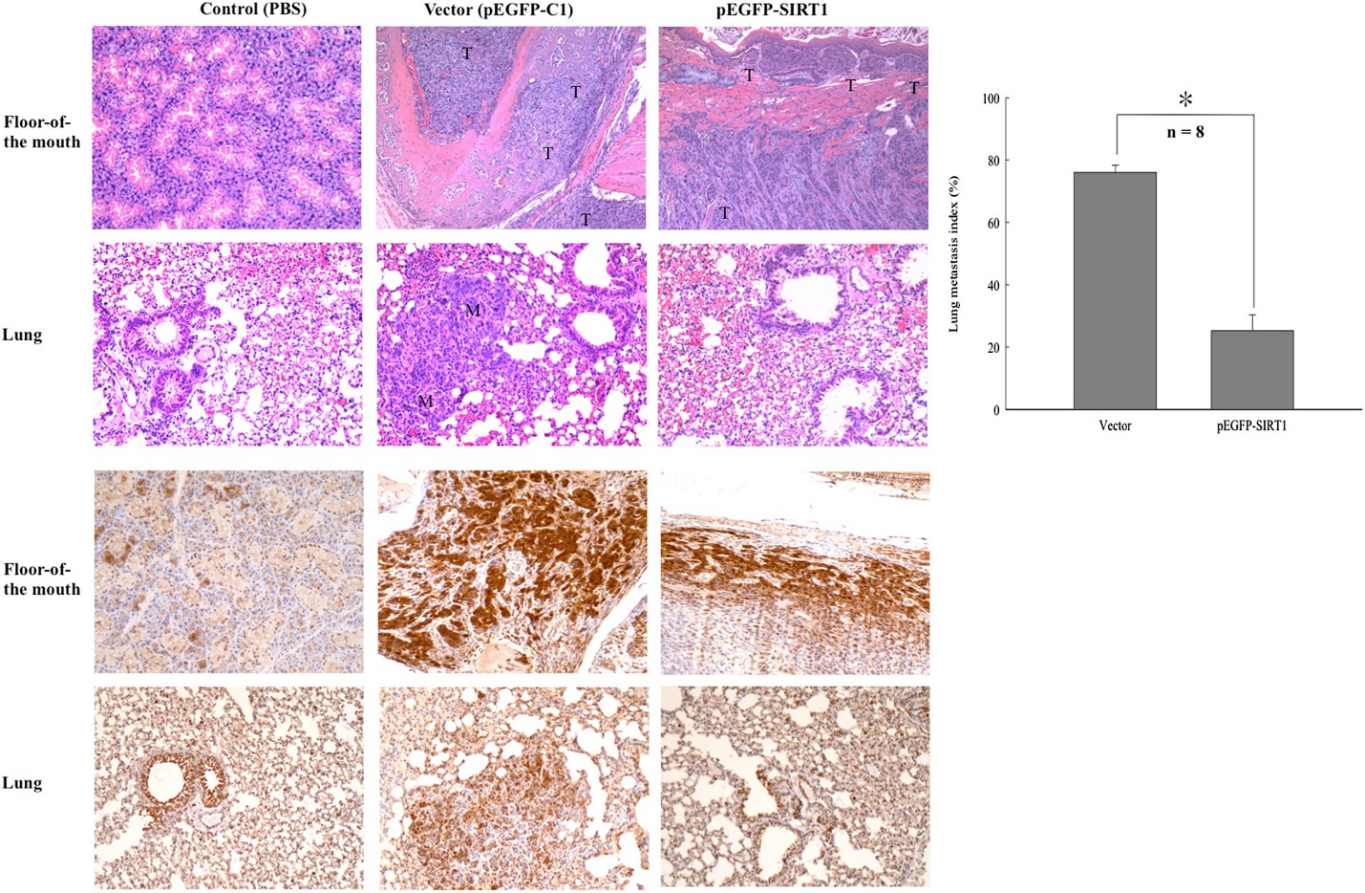
**Overexpression of SIRT1 represses lung metastasis.** SIRT1 inhibited cancer cell metastasis *in vivo* in a floor-of-the mouth murine model with SCID mice. Six-week-old male CB17-SCID mice were anesthetized and injected with human OSCC cell line OECM1-S1 (2.5 × 10^5^ cells/mL) stably expressing SIRT1 expression plasmid or vector alone. All surviving mice were sacrificed after 42 days, and orthotopic tumor and lung tissues were removed, fixed, paraffin-embedded, serially sectioned, and subjected to hematoxylin and eosin (H&E) and immunohistochemical (IHC) staining (magnification, ×100). Upper two panels show results of H&E staining and lower two panels show results of Smad4 IHC staining. The lung metastasis index was calculated as follows: metastatic tumor areas/total lung area. T, tumor tissue; M, metastatic nodule. Quantitative metastasis index is indicated as the mean ± SD. The asterisk indicates as statistically significant difference (*, p <0.05) compared to the control.

Our results showed that SIRT1 inhibits the EMT process in cancer by deacetylating Smad4 and repressing the effect of TGF-β signaling on MMP7. We therefore postulated that overexpression of SIRT1 may suppress cancer cell metastasis *in vivo*. We used a floor-of-the mouth murine model in SCID mice to determine whether SIRT1 inhibits cancer cell metastasis *in vivo*. OECM1 cells were stably transfected with the vector alone (pEGFP-C1) or a vector inducing overexpression of SIRT1 (pEGFP-SIRT1). Ten SCID mice used in the floor-of-the mouth model were injected with OECM1 cells. Two mice were injected with PBS (controls), four were injected with control vector, and four with SIRT1-overexpressing OECM1 cells. As shown in Figure 8, With the exception of PBS control mice, all mice grew similar tumors in the floor-of-the mouth (Figure 8). Upon dissection, the tumors showed multiple foci and poorly differentiated SCCs with prominent lymphovascular invasion at the orthotopic injection site. Among mice injected with vector alone (n = 8) ~75% showed lung metastasis, while ~25% of mice injected with SIRT1-overexpressing vector (n = 8) showed lung metastasis. These results showed that stable overexpression of SIRT1 significantly suppressed lung metastasis of OECM1 cells (p <0.05), resulting in fewer metastatic foci and smaller nodules in the lung. We also examined the tumor region of the extracted tissue by ICH with anti-Smad4 polyclonal antibody, and found higher levels of Smad4 expression in the lung tissue extracted from mice in the vector-only control group. The results indicated that overexpression of SIRT1 in OECM1 cells led to significantly suppressed lung metastasis in the floor-of-the mouth murine model.

## Discussion

**Figure 9:**
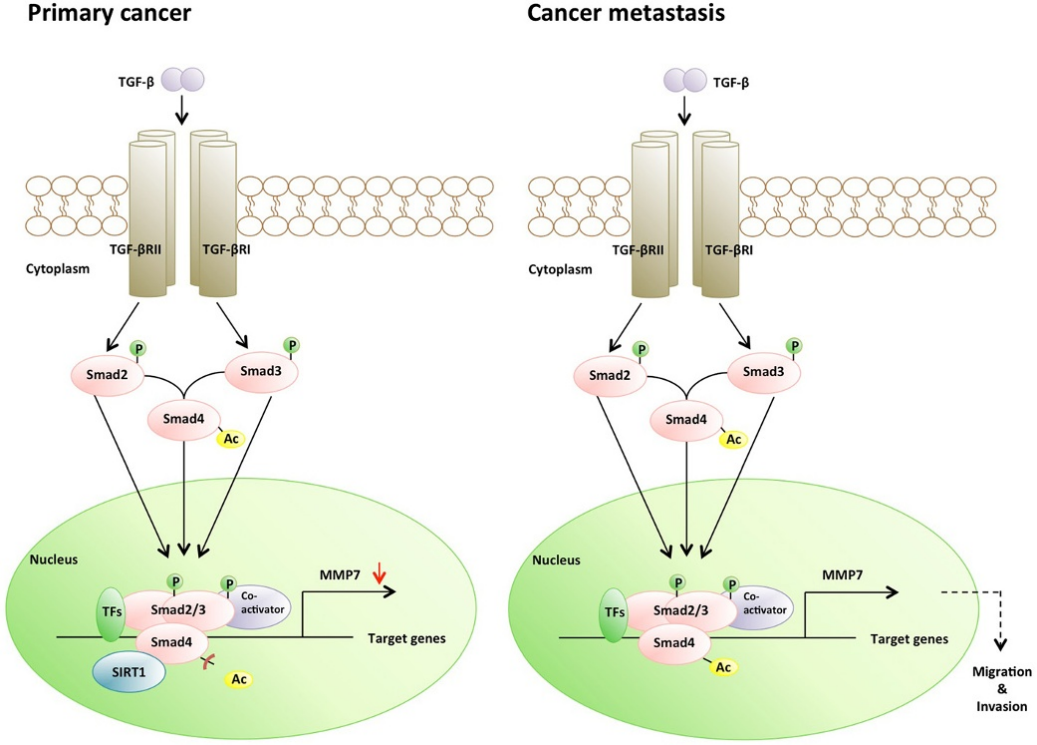
**Schematic representation of SIRT1 effects on metastasis of oral cancer.** SIRT1 reduced cell migration and invasion in oral cancer by deacetylating Smad4 and repressing the effect of TGF-β signaling on MMP7.

In this study, we demonstrated that SIRT1 suppresses the EMT process in oral squamous cell carcinoma cells by deacetylating Smad4 and repressing MMP7 expression. It was previously shown that SIRT1 helps regulate a variety of physiological processes by interacting with nuclear proteins [28, 52–56]. Despite its implied involvement in a variety of physiological processes, the regulatory role of SIRT1 in oral cancer metastasis is poorly understood. In this study, we demonstrated for the first time that SIRT1 is a critical negative regulator of EMT and cell migration *in vitro*, and also of tumor metastasis *in vivo* (immunodeficient mouse model). Our studies showed that compared with expression in HOK cells, SIRT1 was overexpressed in both OSCC cell lines, and a similar result was found in an enzyme activity experiment. We also found that activation of SIRT1 in oral squamous cell carcinoma resulted in decreased cell migration and invasion. Therefore, we propose a molecular mechanism whereby SIRT1 regulates cell migration by interacting with and deacetylating TGF-β-inducing transcription factor Smad4 to suppress MMP7 expression. We found that increased levels of SIRT1 in oral squamous cell carcinoma tissue contributing to decreased Smad4 acetylation and repressed MMP7 activity. In addition, our findings revealed that an absence of SIRT1 led to Smad4 hyperacetylation, MMP7 hyperexpression, and degradation of E-cadherin on the cell surface. These events resulted in release of β-catenin from the E-cadherin-β-catenin complex junctions leading to the nucleus, and promoted metastasis of OSCC cells (Figure 9). In addition to the *in vitro* data showing that up-regulation of SIRT1 led to low cellular invasiveness and migratory abilities, SCID mice with SIRT-overexpressing OSCC cells showed significantly less lung metastasis compared to control mice. The EMT process represents the critical event in the transition from early stage to invasive carcinoma [11, 57], and E-cadherin downregulation is well associated with poor prognosis, lower survival, and higher rates of metastasis in OSCC patients [58–61]. Our results showed that SIRT1 overexpression reduced oral cancer cell migration and metastasis, and these effects were largely independent of any general effects of SIRT1 on oral cancer growth and survival. Taken together, these data suggest that SIRT1 may prevent oral cancer metastasis by blocking the EMT process.

Interestingly, our results differed from previous reports which indicated that SIRT1 serves as a positive regulator of epithelial-mesenchymal transition, the metastatic growth of prostate cancer cells [62], and is associated with malignancy in chronic myelogenous leukemia [63]. Additionally SIRT1 involvement has also been suggested in epigenetic silencing of DNA-hypermethylated tumor suppressor genes in breast cancer cells [64]. Recently, SIRT1 has been shown to be an important target of miR-200 in regulating breast cancer cell migration [65, 66]. Additionally, SIRT1 is highly expressed in various cancers [35, 67] such as prostate cancer, and high levels of SIRT1 expression are associated with a poor prognosis in lung cancer, breast cancer, gastric carcinomas, and B-cell lymphoma [68–71]. In prostate cancer, SIRT1 was shown to enhance cell migration and metastasis by cooperating with EMT transcription factor ZEB1 to suppress E-cadherin transcription. In lung cancer, the SIRT1 activator compound 1720 was shown to increase lung metastasis of implanted breast cancer cells, suggesting SIRT1 as a potential target for suppressing metastasis to the lung [72]. Moreover, miR-200 negatively regulated SIRT1 expression and inhibited the EMT process in normal mouse mammary epithelial cells [65]. However, the role of SIRT1 in tumorigenesis remains controversial, and may depend on the tumor type. A recent report showed that enhanced SIRT1 expression in a β-catenin-dependent mouse model of colon cancer inhibited intestinal-tumor formation, thereby indicating that the effects of SIRT1 might vary in different tumor models, and depend on the presence of appropriate downstream targets [73]. Moreover, SIRT1 was shown to protect against gut carcinomas in APCmin mice [73], as well as inhibit tumorigenesis in p53^+/−^ mice [74, 75]. Wang et al. found that Sirt1^+/−^; p53^+/−^ mice develop tumors in multiple tissues, and activation of SIRT1 by resveratrol reduces tumorigenesis. Moreover, several independent investigations have found reduced levels of SIRT1 in Sirt1^+/−^; p53^+/−^ mice as compared to normal controls, and suggested SIRT1 as an important antagonist of EMT in various types of cancer cells [76–78]. In lung cancer, SIRT1 down-regulation by hypoxia in a SUMOylation-dependent manner promotes EMT, and eventually leads to tumor metastasis. This result supports the hypotheses that SIRT1 activation ameliorates lung cancer metastasis *in vitro* and *in vivo* by blocking the entry of pre-cancerous cells into EMT [76]. Additionally, SIRT1 has been shown to suppress the EMT process in metastasizing breast cancer cells, and the development of fibrosis in organs following their implantation into nude mice. A reduction in SIRT1 levels was shown to promote the metastasis of breast epithelial cells in an orthotopic model of breast cancer, as well as increase the motility of the epithelial cells [78]. Furthermore, while EMT can be induced in both breast and kidney epithelial cells *in vitro*, this induction is repressed by SIRT1 [78]. A previous study found that both miR-520c and miR-373 suppressed SIRT1 mRNA translation, leading to activation of the Ras/Raf/MEK/Erk pathway. Moreover, NF-κB increased MMP9 expression and enhanced the migration of fibrosarcoma cells [79]. Our data builds upon the results in these previous studies by further verifying SIRT1 as a critical regulator of cancer progression, and an important target for prevention or possible treatment of cancer metastasis.

Similar to other cancers, oral cancer metastasis requires degradation of the extracellular matrix via increased expression of matrix metalloproteinases (MMPs). For example, MMP2, 7, and 9 are overexpressed in oral carcinoma tissue [45, 80, 81]. Importantly, MMP7 expression is most pronounced at the invasive front of tumors [45, 82, 83], has been reported as an independent prognostic factor which closely correlates with clinical stage, tumor size, lymph node metastasis, and poor survival of oral cancer patients. Our data also showed that MMP7 expression levels and activity were significantly decreased in OSCC cells overexpressing SIRT1 (Figure 4). Additionally, we found that SIRT1 knockdown OSCC cells showed increased MMP7 secretion and expression. We examined the interaction between SIRT1 and MMP7 in SIRT1 knockdown OSCC cells by immunoprecipitation, and found no direct interaction of SIRT1 with MMP7 (data not shown). A previous study showed that MMP7 was not required for malignant cell invasion in Smad4-deficient adenocarcinomas [84]. Kitamoto et al. found that MMP7 was required for tumor formation, but not for the invasion of the colon cancer cells in which Smad4-dependent TGF-β family signaling had been blocked. Smad4 is indispensable for EMT, and RNA interference-mediated knockdown of Smad4 expression results in preserved E-cadherin expression [20, 85–87]. Additionally, Kume et al. [48] showed that in a mesangial kidney cell line, SIRT1 directly interacted and deacetylated the negative regulator of TGF-β signaling, Smad7, to destabilize the protein. Recently, numerous studies have revealed that TGF-β stimulates the EMT process in certain epithelial cells [22]. TGF-β drives cancer progression by inducing EMT, during which, epithelial cells acquire a mesenchymal phenotype, leading to their enhanced motility and invasiveness. TGF-β signaling directly activates the expression of EMT transcription factors, including **δ**EF1/ZEB1, SIP1/ZEB2, and Snail/SNAI1, which are induced by TGF-β-Smad signaling and play critical roles in TGF-β-induced EMT [88–90]. TGF-β also binds to type II and type I transmembrane kinase receptors, TβRII and TβRI. Following ligand binding, TβRII phosphorylates TβRI, which activates Smad2 and Smad3. These two activated Smad proteins then combine with one Smad4 molecule to form trimeric Smad complexes that translocate into the nucleus and regulate the expression of target genes involved in the EMT process. For example, an active complex formed by Smad3/Smad4 and Snail can bind to the regulatory promoter sequences of genes encoding the epithelial junction proteins E-cadherin and occluding, leading to TGF-β-induced repression of their expression [91]. E-cadherin downregulation decreases the strength of cellular adhesion within a tissue, resulting in increased cellular motility [92]. Furthermore, decreased E cadherin expression during the EMT process is accompanied by increased expression of N-cadherin, which renders a cell more motile and invasive [19–21]. Additionally, TGF-β regulates the expression and activity of extracellular proteases such as matrix metalloproteinases, which allow cells to degrade extracellular matrix proteins and increase their migratory and invasive behaviors. [92–94]. In cancer, epithelial tumor cells become more invasive after undergoing EMT, and access the circulatory system through intravasation, resulting in their dissemination to loci distal from the primary tumor. In our current study, we postulated that the effects of SIRT1 on MMP7 might manifest via its interaction with the TGF-β-activated transcription factor, Smad4. Our data revealed that MMP7 expression levels and activity were significantly decreased in the Smad4 knockdown OSCC cells (Figure 6). Additionally, we used immunoprecipitation techniques to confirm the occurrence of interactions between SIRT1, Smad4, and MMP7 in OSCC cells. Interestingly, SIRT1 was shown to directly interact with Smad4 *in vivo*, but did not interact with MMP7 protein (Figure 5 and Additional file 1). We also showed that overexpression of SIRT1 repressed TGF-β-induced MMP7 expression by deacetylating Smad4, which becomes hyperexpressed and hyperacetylated under conditions of TGF-β stimulation (Figure 5D, 7A and Additional file 2). SIRT1 was shown to affect Smad4 transcriptional activity by deacetylation, and inhibition of Smad4 function repressed TGF-β-induced EMT. These observations clearly show that SIRT1 might influence MMP7 expression, secretion, and activity; and subsequently, cell migration, invasion, and metastasis through Smad4 deacetylation. Furthermore, we also showed that SIRT1-overexpressing cells inhibited MMP7 secretion and increased E-cadherin accumulation, leading to suppression of cellular invasion and migration. Our results indicate that MMPs can mediate both the EMT process [50] and cell metastasis [95], as well as cause nuclear translocation of β-catenin [50, 95] by proteolytic cleavage and release of E-cadherin from the cell surface. It is therefore interesting to speculate that SIRT1 maybe lead to repression of a second pathway involved in EMT, such as the Wnt signaling pathway.

## Conclusions

In conclusion, our study identified SIRT1 as a novel metastatic suppressor which acts through deacetylation of TGF-β-activated transcription factor Smad4 to suppress the effect of TGF-β signaling on MMP7 transcription, leading to reduced migration and metastasis of OSCC cells. SIRT1 shows potential for serving as a predictor and biomarker for metastasis, and up-regulation of SIRT1 is a potentially useful therapeutic strategy for inhibiting the metastasis of oral cancers.

## Methods

### Cell culture and reagents

The HOK cells used in this study were cultured in oral keratinocyte growth medium (ScienCell, Carlsbad, CA, USA) in a 37°C incubator filled with 5% CO_2_, and were routinely passaged at 90% confluence. Five human OSCC cell lines [HSC-3 (tongue carcinoma, Japan Health Science Research Resources Bank, JRCB 0623), OECM-1 (Gingival carcinoma) [96], OC3 (Buccal mucosa) [97], SCC4 (tongue carcinoma, American Type Culture Collection, ATCC**®** CRL1624™), and SCC 25 (ATCC**®** CRL1628™)] were used in this study. HSC-3 and OC3 cells were cultured in Dulbecco’s modified Eagle’s (DMEM) medium containing 2 mM glutamine. OECM-1 cells were maintained in RPMI 1640 medium, while SCC4 and SCC25 cells were cultured in DMEM/F12 medium. Each culture medium was supplemented with 10% fetal bovine serum and 100 units/mL each of penicillin and streptomycin (Invitrogen, Camarillo, CA, USA). All OSCC cells were maintained at 37°C in a humidified atmosphere of 5% CO_2_. The SIRT1 agonist (resveratrol; RSV) and antagonists (nicotinamide and sirtinol) were purchased from Sigma-Aldrich (St. Louis, MO, USA).

### Plasmid construction and transient transfection

The human SIRT1 coding region (GeneBank: NM_012238) was amplified by polymerase chain reaction (PCR) using the forward primer 5′-GTCGACATGGCGGACGAGGCGGCCCTCGC-3′ to introduce a SalI site, and 5′-GGATCCCTATGATTTGTTTGATGGATAGTTC-3′ to introduce a BamHI site. The conditions for PCR were as follows: denaturing for 30 sec at 94°C, annealing for 30 sec at 62°C and elongation for 1 minute at 72°C for 35 cycles. The full-length of SIRT1 gene was subcloned into the constitutive mammalian expression vector pEGFP-C1 (GeneDireX, Las Vegas, NV, USA), and transfection was verified by DNA sequencing. Transfected cells were seeded in 6-cm diameter dishes at 5 × 10^5^ cells/dish, and transfected with either pEGFP-SIRT1 or empty vector using Lipofectamine 2000 (Invitrogen), according to the manufacturer′s protocol. Transfected cells were further examined in cell proliferation assays.

OECM1 cells were transiently transfected with small interfering RNA (siRNA) (150 nM; a pool of 3 target-specific siRNAs) against SIRT1, or with a nontargeting control (GeneDireX) in Opti-MEM**®** I reduced serum medium containing Lipofectamine RNAiMAX (Invitrogen). Transfection efficiency was assessed by western blot.

### RNA isolation and quantitative real-time PCR

For gene expression analysis, pairs of tumor and normal marginal tissues were obtained from 21 OSCCs. The tissues were frozen and stored in liquid nitrogen at −196°C until use. Total RNA obtained from cultured cells and human tissue was extracted using TRIzol reagent (Invitrogen). cDNA was then reverse-transcribed and amplified by PCR using a Transcriptor First Strand cDNA Synthesis kit (Roche Diagnostics, Mannheim, Germany). Quantitative RT-PCR was jperformed using the FastStart Universal SYBR Green Master mix (Roche) and an Applied Biosystems ABI 7900 RealTime PCR System (Applied Biosystems, Foster City, CA, USA). The oligonucleotide primers used for human SIRT1, Smad4, MMP7, and glyceraldehyde-3-phosphate dehydrogenase (GAPDH) were as follows: SIRT1-F 5′-TAGCCTTGTCAGATAAGGAAGGA-3′; SIRT1-R 5′-ACAGCTTCACAGTCAACTTTGT-3′; Smad4-F 5′-CTCATGTGATCTATGCCCGTC-3′; Smad4-R 5′-AGGTGATACAACTCGTTCGTAGT-3′; MMP7-F 5′-TCCAACCTATGGAAATGGAGA-3′; MMP7-R 5′-GGAGTGGAGGAACAGTGCTT-3′; GAPDH-F 5′-GAGTCAACGGATTTGGTCGT-3′; GAPDH-R 5′-GACAAGCTTCCCGTTCTCAG-3′.

Gene expression levels were normalized using GAPDH as an internal reference gene, and the average relative change was calculated from triplicate or quintuplicate determinations made by relative quantification, and applying the delta-delta cycle threshold method. The protocol for this study was approved by the Institutional Review Board (IRB) of the Department of Oral and Maxillofacial Surgery of Chi-Mei Medical, Liouying, Taiwan (EC-1000202-R1).

### Cell chemotactic migration and invasion assay

The chemotactic migration of cells was evaluated using a 24-well chemotaxis chamber equipped with 8-μm pore size membranes (Becton-Dickinson labware, Franklin Lakes, NJ, USA). Cell invasion ability was assessed using Falcon Cell Culture Inserts with Matrigel (BD). Samples containing 1 × 10^5^ cells were resuspended in serum-free medium with 0.1% BSA (Sigma-Aldrich), and then plated onto the transwell chamber. The chambers were incubated for 24 h with complete culture medium added in the lower chamber. Non-mobile cells were removed, and the chambers were stained with crystal violet. Photomicrographs of 5 regions were captured from duplicated chambers. The numbers of cells were counted and normalized to the control. All experiments were performed in triplicate and repeated three times.

### Cytosolic, nuclear isolation and immunoprecipitation

Cytosolic and nuclear extracts were prepared using a NE-PER Nuclear and Cytoplasmic Extraction Reagents kit (Thermo Fisher Scientific, Basingstoke, UK) following the manufacturer’s protocol. Isolated nuclear extracts were lysed with RIPA buffer, and then subjected to direct western blot analysis or immunoprecipitation. Then, 2 mg of protein from each sample (total lysate or nuclear extract) was used for immunoprecipitation with a Pierce® Crosslink IP Kit (Pierce), and the results were analyzed by western blot.

### Western blot analysis

Cells were lysed directly in RIPA buffer containing 50 mM Tris–HCl, pH 7.8 150 mM NaCl, 5 mM EDTA, 5 μL/mL Triton X-100, 5 μL/mL Nonidet-P40, 1 μL/mL sodium deoxycholate, and an EDTA-free complete protease inhibitor cocktail (Roche; Basel, Switzerland) on ice for 30 minutes. The lysates were adjusted for protein concentration with a BCA protein assay kit (Bio-Rad, Hercules, CA, USA). The lysate proteins were resolved by 10% SDS-PAGE and then transferred to PVDF membranes. The membranes were blocked and incubated with specific antibodies against SIRT1, E-cadherin, vimentin, acetylated-lysine (Cell Signaling Technology, Beverly, MA, USA), actin (Sigma-Aldrich, St. Louis, USA), N-cadherin (H63), Smad4, MMP7, and GAPDH (Santa Cruz Biotechnology, Santa Cruz, CA, USA). The resolved protein bands were visualized by enhanced chemiluminescence ECL-Plus detection system (Perkin Elmer-NEN, France).

### Immunohistochemistry

IHC was conducted to detect protein expression in paraffin-embedded oral squamous cell carcinoma specimens. The slides were stained with rabbit anti-SIRT1 polyclonal antibody (1:25 Santa Cruz Biotechnology, Santa Cruz, CA, USA) and goat anti-Smad4 polyclonal antibody (1:100, Santa Cruz Biotechnology) using an automatic slide stainer BenchMark XT (Ventana Medical Systems; Tucson AZ, USA). Hematoxylin was used as the counterstain. Two independent pathologists evaluated each slide under a light microscope. Immunoreactivity was classified by estimating the percentage (P) of tumor cells exhibiting characteristic staining (from an undetectable level, 0%, to homogeneous staining, 100%) and by estimating the intensity (I) of staining (1, weak staining; 2, moderate staining; 3, strong staining). Results were scored by multiplying the percentage of positive cells by the intensity (i.e. quick score Q = P x I; maximum = 300) [98].

### *In vivo*metastasis assay

Six-week-old male CB17-SCID mice (weights 20-25 g) were anesthetized by intraperitoneal injection with 100 mg/kg ketamine and 10 mg/xylazine. Prior to injection, human OSCC cell line OECM1-S1 stably expressing SIRT1 expression plasmid or vector alone was grown to 70% confluence. The OSCC cells were suspended in RPMI-1640, chilled on ice, and adjusted to a final concentration of 2.5 × 10^5^ cells/mL. For detecting metastasis, we used an orthotopic floor-of-the mouth murine model which was monitored for 28 to 42 days. After sacrifice, the organ and tissues were removed, fixed, paraffin-embedded, serially sectioned, and subjected to hematoxylin and eosin (H&E) and IHC staining.

### Enzyme activity assay

SIRT1 proteins obtained from total lysates of cultured cells and human tissue were concentrated using a Pierce® Crosslink IP Kit (Pierce), according to the manufacturer’s recommendations. Protein concentrations were determined using a Bio-Rad protein assay kit (Bio-Rad; Hercules, CA, USA). SIRT1 enzyme activity was determined using a SIRT1 Fluorometric Kit (Biomol International LP, Plymouth Meeting, PA, USA) according to the manufacturer’s instructions. This assay uses a small lysine-acetylated peptide, corresponding to K382 of human p53, as a substrate. The lysine residue is deacetylated by SIRT1, and this process is dependent on addition of exogenous NAD^+^. The fluorescence values obtained in the absence of NAD^+^ did not differ from those obtained with the blank. Addition of exogenous NAD^+^ was necessary, and this was most likely because endogenous NAD^+^ was lost during sample preparation. The enzyme activity assay for SIRT1 was performed in 50 μL of reaction buffer (Biomol International, BML-KI286) containing 25 μL of SIRT1 proteins (10 ng/μL), 50 μM Fluor de Lys–SIRT1 substrate, and 500 μM NAD^+^. Deacetylation reactions were conducted at 37°C for 60 minutes, and stopped by adding 50 μL of stop solution made by combining Fluor de Lys Developer (Enzo Life Sciences, Farmingdale, NY, USA) and 2 mM nicotinamide, followed by incubation at 37°C for 1 h. Enzyme activity was determined by spectrophotometric readings made at excitation and emission wavelengths of 360 nm and 460 nm, respectively, in endpoint mode, using a SpectraMax M2 microplate reader (Molecular Devices Corporation, Sunnyvale, CA, USA). Calculations of net fluorescence were made after subtracting values for a blank consisting of buffer without NAD^+^.

### MMP7 ELISA and Casein zymography

Total MMP7 concentrations in OSCC cells were assessed using the Quantikine Human MMP7 Immunoassay Kit (R&D Systems, Minneapolis, MN, USA) according to the manufacturer’s instructions. For casein zymography, total proteins were loaded on precast 12% Novex zymogram blue casein gels (Invitrogen, Carlsbad, CA, USA) to measure MMP7 proteolytic activity. Following electrophoresis, the gels were renatured in Novex Zymogram Renaturing Buffer for 30 minutes at room temperature, and then incubated at 37°C in Novex Zymogram Developing Buffer (Invitrogen) to permit degradation of substrate in the gel matrix. Enzymatic activity was visualized as a clear band against a blue background [99, 100].

### Statistical analysis

All data are reported as the mean value ± S.D. obtained from at least 3 independent experiments. The statistical significance of differences between means was assessed by ANOVA. The P values for linear trends of mRNA expression were analyzed using the t test (slope estimate) in simple linear regression models. P-values <0.05 and 0.01 (depending on the experiment) were considered statistically significant.

## Electronic supplementary material

Additional file 1: Figure S1: SIRT1 deacetylates Smad4 in HSC3 cell lines. (A) Ectopic expression of SIRT1 reduces acetylation levels of Smad4. Equal amounts of proteins (20 ug) from the HSC3 cells were transient transfected with pEGFP-SIRT1 or vector alone (pEGFP-C1) for 0–48 h and analyzed by Western blot. (B) Western blotting reveal the expression and acetylation levels of endogenous Smad4 in HSC3 cell lines were transient transfected with pEGFP-SIRT1 or vector alone (pEGFP-C1) for 24 h, and were treated with TGF-β 5 ng/ml for 48 h. Western blots were probed with SIRT1, acetylated-Lysine (Ac-K) and Smad4. (TIFF 8 MB)

Additional file 2: Figure S2: SIRT1 represses the expression of MMP-7 by deacetylating Smad4 in HSC3 cell lines. (A) Western blotting reveal the expression levels of Smad4, MMP-7 and E-cadherin in HSC3 cell lines were transient transfected with pEGFP-SIRT1 or vector alone (pEGFP-C1) for 24 h, and were treated with TGF-β 5 ng/ml for 48 h. (B) MMP-7 activities of SIRT1-overexpressing or mock-transfected HSC3 cells were assayed by casein zymography after treatment with or without TGF-β 5 ng/ml for 48 h. (TIFF 8 MB)

## Abbreviations

SIRT: Sirtuin
HOK: Human oral keratinocyte
OSCC: Oral squamous cell carcinoma
MMP: Matrix metalloproteinase
NAD: Nicotinamide adenine dinucleotide
TGF-β: Transforming-growth-factor-β
GAPDH: Glyceraldehyde-3-phos-phate dehydrogenase
RSV: Resveratrol.
