# Supplementary figures and images for: Role of SIRT1 in regulation of epithelial-to-mesenchymal transition in oral squamous cell carcinoma metastasis
Source: Mol Cancer. 2014 Nov 26;13:254. doi: 10.1186/1476-4598-13-254 (PMC4258025; doi:10.1186/1476-4598-13-254)

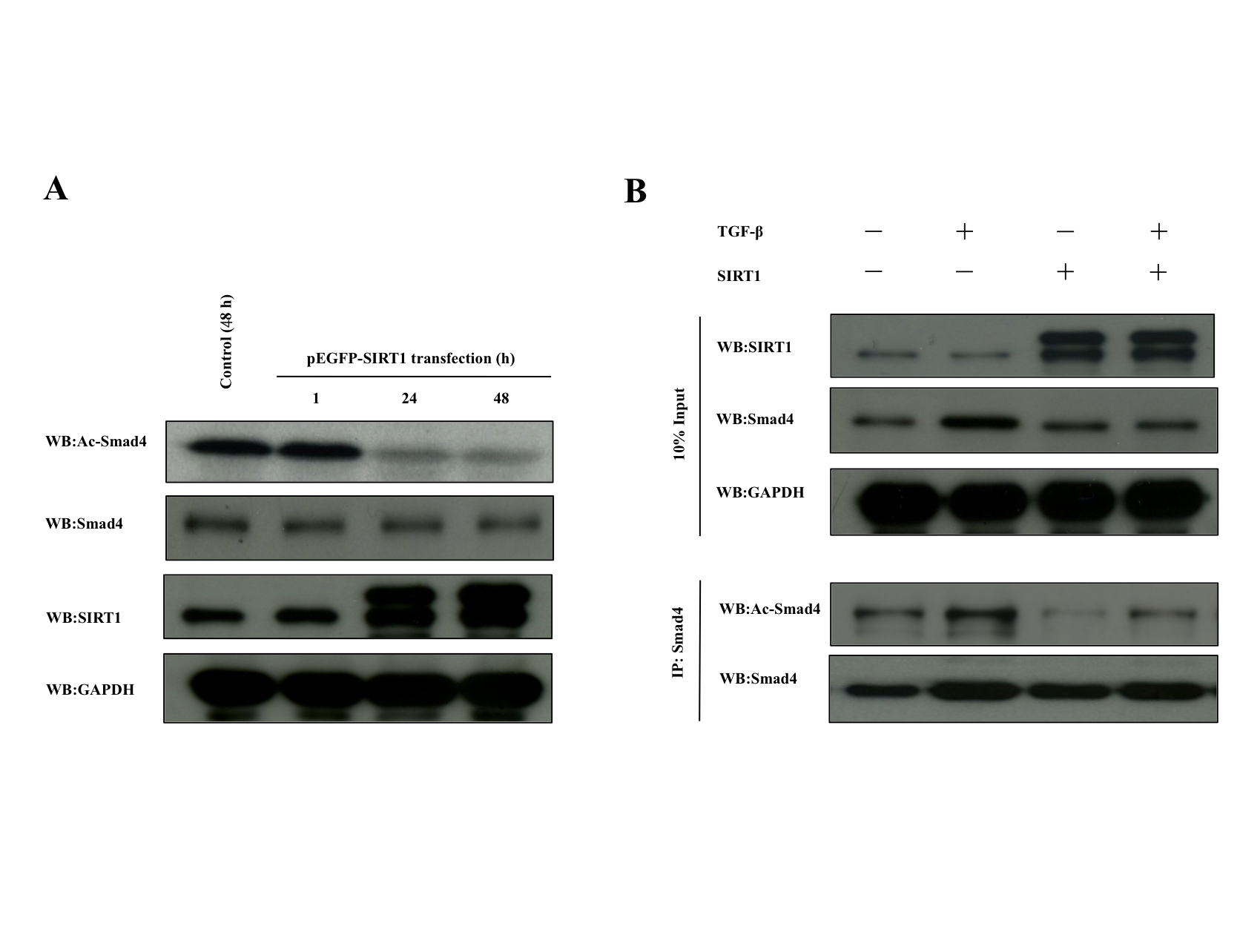

Supplement: Supplementary file 1 — Additional file 1: Figure S1: SIRT1 deacetylates Smad4 in HSC3 cell lines. (A) Ectopic expression of SIRT1 reduces acetylation levels of Smad4. Equal amounts of proteins (20 ug) from the HSC3 cells were transient transfected with pEGFP-SIRT1 or vector alone (pEGFP-C1) for 0–48 h and analyzed by Western blot. (B) Western blotting reveal the expression and acetylation levels of endogenous Smad4 in HSC3 cell lines were transient transfected with pEGFP-SIRT1 or vector alone (pEGFP-C1) for 24 h, and were treated with TGF-β 5 ng/ml for 48 h. Western blots were probed with SIRT1, acetylated-Lysine (Ac-K) and Smad4. (TIFF 8 MB) [file 12943_2014_1453_MOESM1_ESM.tiff]

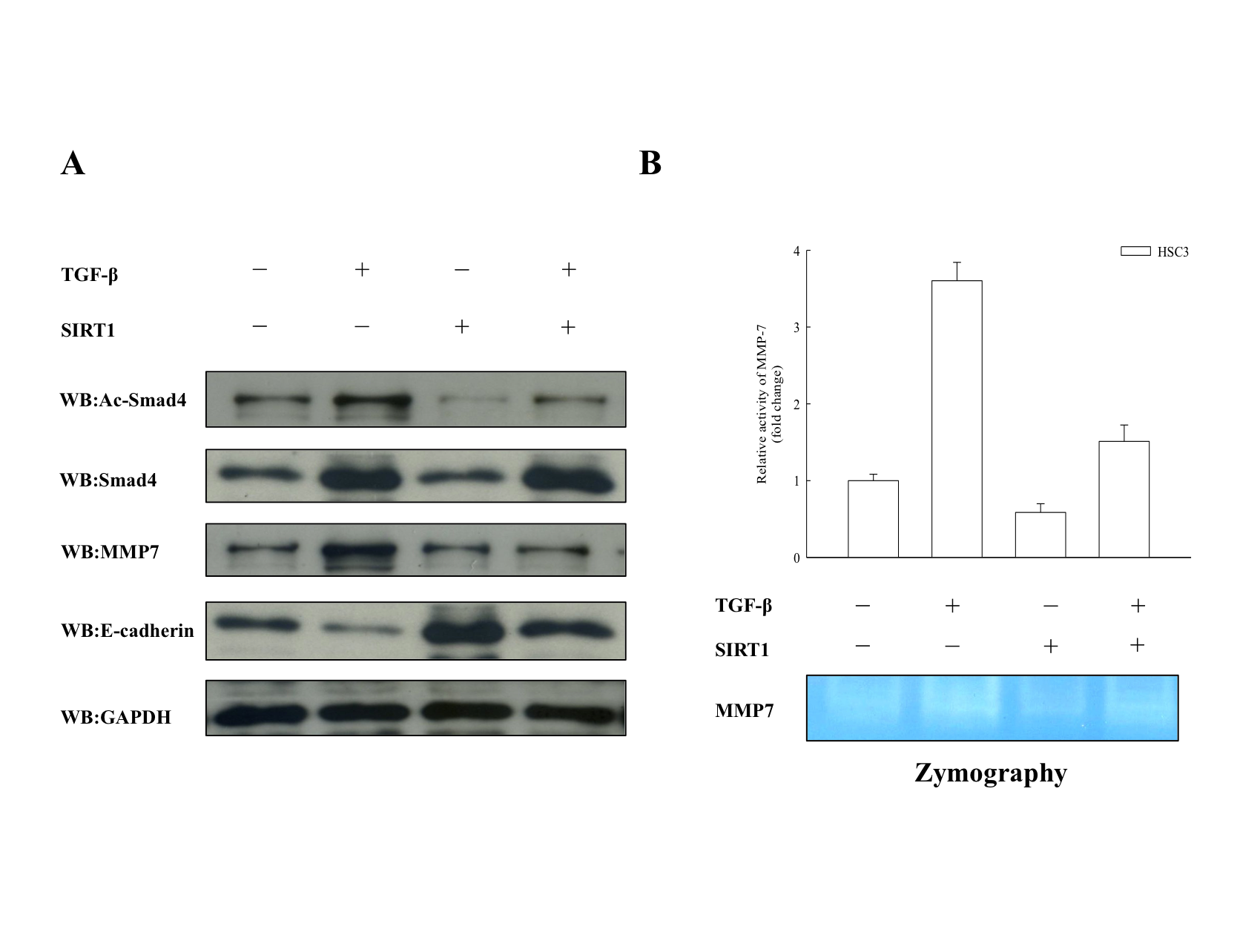

Supplement: Supplementary file 2 — Additional file 2: Figure S2: SIRT1 represses the expression of MMP-7 by deacetylating Smad4 in HSC3 cell lines. (A) Western blotting reveal the expression levels of Smad4, MMP-7 and E-cadherin in HSC3 cell lines were transient transfected with pEGFP-SIRT1 or vector alone (pEGFP-C1) for 24 h, and were treated with TGF-β 5 ng/ml for 48 h. (B) MMP-7 activities of SIRT1-overexpressing or mock-transfected HSC3 cells were assayed by casein zymography after treatment with or without TGF-β 5 ng/ml for 48 h. (TIFF 8 MB) [file 12943_2014_1453_MOESM2_ESM.tiff]
